# Sarcoid reaction in eyebrow tattooing: a complication of a common cosmetic procedure^☆^

**DOI:** 10.1016/j.abd.2021.08.019

**Published:** 2023-04-27

**Authors:** Tamires Ferri Macedo, Simone Perazzoli, Renan Rangel Bonamigo, Renata Heck

**Affiliations:** Service of Dermatology, Sanitary Dermatology Outpatient Clinic, Secretaria de Saúde do Estado do Rio Grande do Sul, Porto Alegre, RS, Brazil

Dear Editor,

In the last decades, facial pigmentation techniques for aesthetic purposes have become common. Among them is eyebrow tattooing or micropigmentation. Unlike traditional tattoos, in which the pigment is deposited in the deeper layers of the dermis, in micropigmentation, the semi-permanent pigment is deposited in the upper layer of the dermis. Adverse reactions to this technique include infection, contact dermatitis, granulomatous reactions, and Koebner phenomenon.^1^

A 30-year-old female patient, with a history of previous bariatric surgery, without other comorbidities, complained of raised eyebrows for three months. She had repeatedly undergone micropigmentation of the region over the past four years, the last being 14 months before. She denied any systemic symptoms. On examination, she had raised plaques on the topography of the eyebrows, especially on the right, in addition to areas of alopecia (Fig. 1A). At dermoscopy, homogeneous orange-brown areas and rarefied hairs were seen (Fig. 2). The remainder of the physical examination was normal. Complementary tests including serum calcium level, electrocardiogram, chest X-ray, serum protein electrophoresis, and tuberculin skin test were normal.

**Figure 1:**
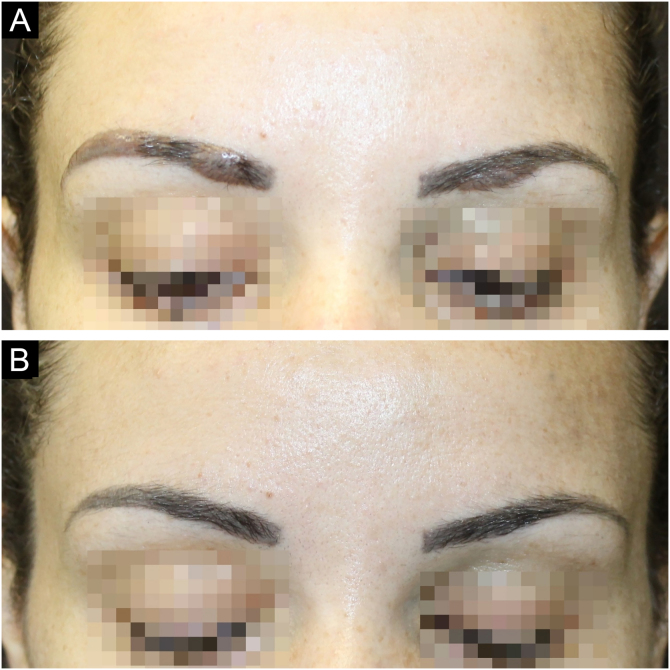
(A) Infiltrated and well-defined plaques on the topography of both eyebrows; rarefied hairs. (B) Complete regression of the lesions with eyebrow regrowth

**Figure 2:**
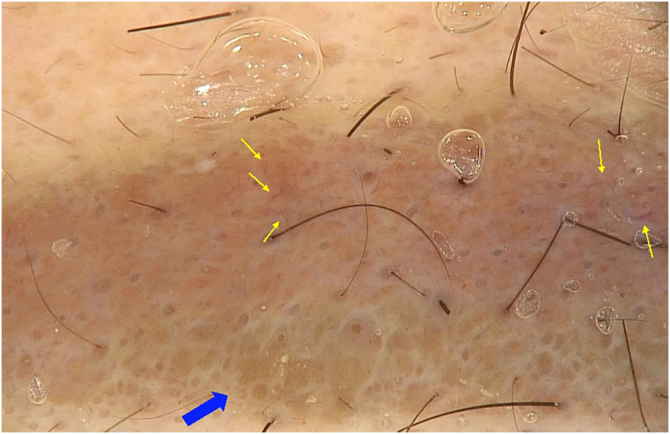
Dermoscopy (×20) showing area of homogeneous orange-brown color and rarefied hairs (blue arrow) and linear vessels (yellow arrows)

Histopathology showed non-caseating chronic granulomatous dermatitis with a sarcoid pattern (Fig. 3); acid-fast bacillus (AFB) and fungal tests were negative. With the diagnosis of sarcoid reaction secondary to the tattooing of the eyebrows, therapy with doxycycline 100 mg/day and fludroxycortide occlusive treatment was performed for 15 days. The patient missed the reassessment appointment and returned after three months with complete regression of the lesions (Fig. 1B). She was advised not to repeat the procedure.

**Figure 3:**
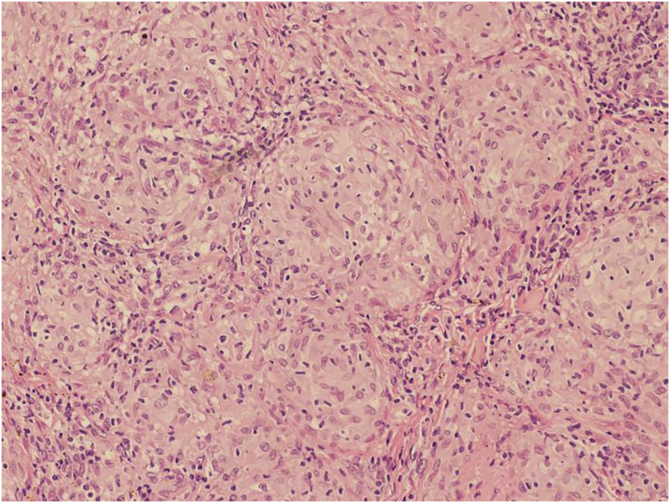
Histopathology showing non-caseating granulomas with a sarcoid pattern (Hematoxylin & eosin, ×50)

Micropigmentation is normally performed with a portable tattoo pen, which is smaller than the traditional tattoo device. Ready-made paints are available on the market, but some professionals make their own mixtures. Pigment washout may occur during the first few days of healing, then the remaining pigment particles are stored in dermal macrophages and fibroblasts.^2^, ^3^

Sarcoid granulomas can develop in areas of tattooing or permanent makeup as isolated reactions or as part of systemic sarcoidosis. The time between the tattoing and the reaction onset is variable, and there may be a long latency period, justifying the investigation of systemic sarcoidosis.^1^, ^2^

Topical and intralesional corticosteroids are the first line of treatment.^1^ Studies suggest that tetracyclines inhibit granuloma formation, and their role in the treatment of sarcoid reactions has been documented.^4^ There are also reports of systemic treatment with allopurinol and antimalarials.^5^ The patient had an excellent response after a short period of treatment with occlusive corticosteroids associated with doxycycline, with no lesion recurrence to date.

While numerous cases of sarcoid granulomas have been reported in body tattoos, few have been related to eyebrow micropigmentation. With the greater prevalence of this cosmetic technique, it is important to recognize the possible adverse reactions, as well as the adequate management. Moreover, it is crucial to remember the importance of investigating systemic sarcoidosis in these patients.

## Financial support

None declared.

## Authors' contributions

Tamires Ferri Macedo: Approval of the final version of the manuscript; drafting and editing of the manuscript; collection, analysis, and interpretation of data; critical review of the literature.

Simone Perazzoli: approval of the final version of the manuscript; drafting and editing of the manuscript; collection, analysis, and interpretation of data; critical review of the literature.

Renan Rangel Bonamigo: Approval of the final version of the manuscript; design and planning of the study; intellectual participation in the propaedeutic and/or therapeutic conduct of the studied cases; critical review of the manuscript.

Renata Heck: Approval of the final version of the manuscript; design and planning of the study; effective participation in research orientation; intellectual participation in the propaedeutic and/or therapeutic conduct of the studied cases; critical review of the manuscript.

## Conflicts of interest

None declared.
